# Cord Bilirubin and 24-Hour Serum Bilirubin as Predictors of Hyperbilirubinemia in Term Appropriate-for-Gestational-Age (AGA) Neonates

**DOI:** 10.7759/cureus.105278

**Published:** 2026-03-15

**Authors:** Divyasri Sabbu, Sudhakar Chiluka, K Raghunath Reddy, G Sudhakar Babu

**Affiliations:** 1 Department of Pediatrics, Employees' State Insurance Corporation (ESIC) Medical College, Sanathnagar, IND

**Keywords:** 24-hour bilirubin, cord blood bilirubin, hyperbilirubinemia, neonatal jaundice, prediction

## Abstract

Background: Jaundice is common in neonates, yet clinically significant hyperbilirubinemia may develop after early discharge. Bilirubin typically peaks between days 3 and 5; therefore, reliable predischarge biomarkers could improve risk stratification and follow-up planning. This study assessed whether cord blood total serum bilirubin (TSB) and 24-hour TSB can predict significant hyperbilirubinemia on day 5 in healthy term neonates.

Methods: This hospital-based prospective observational study enrolled 200 healthy inborn term neonates (gestational age ≥ 37 weeks, birth weight ≥ 2.5 kg) and followed them from birth to day 5. Neonates with sepsis, asphyxia, intrauterine growth restriction, congenital anomalies, or ABO/Rh incompatibility were excluded. Cord bilirubin was measured at birth and venous bilirubin at 24 hours. Day-5 TSB was measured at approximately 120 hours (±6 hours). The prespecified analytic outcome for prediction was day-5 TSB ≥ 15 mg/dL. Phototherapy, when clinically required, was initiated according to the American Academy of Pediatrics (AAP) 2022 hour-specific phototherapy thresholds for infants ≥35 weeks and was recorded as a clinical management outcome. Receiver operating characteristic (ROC) analysis was used to assess discrimination and determine optimal cutoff values.

Results: Using the prespecified analytic endpoint of day-5 TSB ≥15 mg/dL, 77/200 neonates (38.5%) met the study outcome. Cord bilirubin showed strong discrimination (AUC 0.893, 95% CI 0.846-0.940; p < 0.001); a cutoff of 1.75 mg/dL yielded 89.7% sensitivity and 69.9% specificity. The 24-hour bilirubin also performed well (AUC 0.880, 95% CI 0.83-0.93; p < 0.001); a cutoff of 5.80 mg/dL provided 88.0% sensitivity and 80.5% specificity, with 54.0% positive predictive value and 96.0% negative predictive value. Lower birth weight (2.5-3.0 kg) was significantly associated with the outcome (p < 0.001), whereas sex was not (p = 0.57).

Conclusion: Cord bilirubin and 24-hour bilirubin both predict significant day-5 hyperbilirubinemia with high discrimination. The high negative predictive value at 24 hours supports early identification of low-risk neonates, while elevated values at either time point justify closer follow-up rather than automatic treatment. Using two early measurement time points may strengthen discharge counseling and planned surveillance; however, these cutoff values should be used as screening tools rather than treatment thresholds.

## Introduction

Many term neonates develop a physiological rise in bilirubin during the first week of life and improve without intervention [1,2]. Nevertheless, a smaller but clinically important proportion progresses to bilirubin levels that meet treatment thresholds after discharge. This post-discharge period is particularly relevant because infants may appear clinically well during the first 24 hours while feeding is still being established, and discharge often occurs before bilirubin reaches its usual peak. When scheduled follow-up is delayed or inconsistent, the bilirubin burden may become substantial before poor feeding or excessive sleepiness is recognized. Consequently, by the time these symptoms appear, hyperbilirubinemia may already be significant and more difficult to reverse. The practical aim in neonatal care is therefore to prevent severe hyperbilirubinemia before neurological risk emerges.

Clinical assessment alone does not reliably identify infants at risk. Visual estimation varies with skin tone, ambient lighting, and inter-examiner judgment [3,4]. Predischarge clinical zoning can assist early orientation, but it cannot independently support safe discharge decisions. Objective, time-linked bilirubin measurement provides more robust risk stratification. In this context, the hour-specific predischarge bilirubin nomogram represented an important advance by aligning bilirubin values with post-discharge risk classification and follow-up planning [5].

However, in many health systems, post-discharge surveillance remains inconsistent, and missed follow-up contributes to delayed recognition of clinically significant rises. Risk prediction therefore needs to begin earlier and rely on measurements that are feasible within routine care pathways. Cord blood sampling is available at birth without additional invasive procedures, and a 24-hour bilirubin measurement aligns with early neonatal review. If these two time points can anticipate day-5 risk, follow-up intensity can be calibrated according to biological risk rather than applied uniformly.

Current evidence supports this approach. Cord bilirubin-based screening has identified higher-risk neonates across multiple cohorts, although diagnostic performance varies and some thresholds demonstrate stronger rule-in than rule-out characteristics [6]. Stratified analyses consistently show increasing subsequent jaundice burden across rising cord bilirubin categories, with reasonable discrimination for phototherapy requirement in selected populations [7,8]. Meta-analytic evidence suggests pragmatic cord bilirubin cutoffs for closer monitoring, while also highlighting variability in sensitivity across settings and population profiles [9]. A similar rationale applies to early 24-hour bilirubin measurement, which has demonstrated strong rule-out performance, including high negative predictive value at day-1 thresholds near 6 mg/dL in several reports [10-12].

Despite these advances, late post-discharge bilirubin peaks may still be underdetected, and single biomarkers generally perform best as screening tools rather than stand-alone triggers for therapy. A dual time-point strategy is therefore biologically plausible and clinically pragmatic. Cord bilirubin reflects the baseline bilirubin load at birth, whereas the 24-hour value reflects the early postnatal trajectory shaped by bilirubin production, hepatic conjugation capacity, and feeding transition. Together, these measurements may provide a more stable estimate of which infants are likely to exceed a clinically meaningful day-5 threshold.

Accordingly, this study evaluated whether cord blood total serum bilirubin (TSB) and 24-hour TSB can predict significant hyperbilirubinemia on day 5 in healthy term neonates with gestational age ≥ 37 weeks and birth weight ≥ 2.5 kg. Within this restricted clinical population, early bilirubin measurements may help distinguish infants at lower versus higher predicted risk of day-5 hyperbilirubinemia and thereby guide the intensity of follow-up and discharge counseling. These findings apply to term neonates similar to those studied and should not be extrapolated to preterm infants, low-birth-weight neonates, hemolytic jaundice, or other high-risk clinical groups.

## Materials and methods

Study design

A hospital-based prospective observational study was conducted, with follow-up from birth to day 5 of life. The study evaluated whether bilirubin measured at two early time points (cord blood at birth and venous blood at 24 hours) predicts clinically significant hyperbilirubinemia during the usual post-discharge peak period. Cord blood TSB was considered a baseline measure, whereas 24-hour TSB was considered an early trajectory marker. The primary analytic outcome was significant hyperbilirubinemia on day 5, prespecified as venous TSB ≥ 15 mg/dL measured at approximately 120 hours (allowable window ±6 hours). Phototherapy decisions followed the American Academy of Pediatrics (AAP) 2022 hour-specific thresholds and were recorded separately as a clinical management outcome [13].

Study setting and duration

The study was conducted over a one-year period in the postnatal care ward of the Department of Pediatrics, Employees' State Insurance Corporation (ESIC) Medical College and Hospital, Sanathnagar, Hyderabad. Only inborn neonates were included to ensure accurate sampling times and consistent clinical surveillance.

Study population and recruitment

Healthy term neonates roomed-in with their mothers were enrolled by consecutive sampling during the study period. Enrollment occurred soon after birth. Each neonate underwent daily clinical assessment until day 5 of life. If discharge occurred before day 5, caregivers were instructed to return for a scheduled day-5 visit for outcome sampling.

Sample Size Estimation

Sample size was estimated using Cochran’s formula for a single proportion, assuming a prevalence of significant hyperbilirubinemia of 12%, 95% confidence level, and absolute precision of 5%.

$n = \frac{z^{2}\times p\times \left( 1-p \right)}{d^{2}}$

$n = \frac{\left( 1.96 \right)^{2}\times 0.12\times 0.88}{\left( 0.05 \right)^{2}}$

$n = 162$

To account for potential loss to follow-up and incomplete sampling, the calculated sample size was increased by approximately 15%. A final sample size of 200 neonates was planned to ensure an adequate number of outcome events for receiver operating characteristic (ROC) analysis and cutoff estimation.

Eligibility criteria

Term neonates with gestational age more than 37 weeks, assessed using the New Ballard Score (NBS) [14] and supported by obstetric dating when available, birth weight more than 2.5 kg, and inborn neonates roomed-in with the mother and clinically stable at enrollment were included in the study.

Exclusion criteria included clinical suspicion or diagnosis of neonatal sepsis at enrollment or during follow-up; birth asphyxia or significant perinatal depression as per unit criteria; intrauterine growth restriction; major congenital anomalies; ABO or Rh blood group incompatibility or other evidence of immune hemolysis.

Baseline data collection

Neonatal variables recorded at birth included gestational age, birth weight, sex, mode of delivery, and Apgar scores at one and five minutes. Maternal data included blood group and Rh status and relevant perinatal history related to jaundice risk and feeding transition. Neonatal blood grouping was performed as per routine ward practice.

Clinical assessment and follow-up

A structured clinical assessment was performed on day 1 and then daily until day 5. Monitoring included temperature, heart rate, respiratory rate, perfusion, hydration status, activity, and feeding adequacy. Urine output and stool pattern were documented. The neonate was examined for pallor, bruising, cephalohematoma, and other birth-related trauma. Jaundice severity was assessed daily using Kramer dermal zones under consistent ward lighting where feasible. Neonates developing poor feeding, lethargy, or rapidly progressive jaundice underwent additional clinical review and bilirubin testing as clinically indicated. Clinical care decisions were not modified for study purposes.

Blood sampling and bilirubin estimation

Cord Blood Sampling

Cord blood was collected immediately after delivery from the placental end of the umbilical cord using an aseptic technique. Approximately 1-2 mL of blood was collected, protected from direct light, and transported promptly to the laboratory. Total bilirubin was measured and reported in milligrams per deciliter (mg/dL) using the hospital standard laboratory method.

24-Hour Venous Sampling

A peripheral venous sample of 1-2 mL was obtained at 24 hours of life, with an allowable window of ±4 hours. TSB was measured using the same laboratory method and reported in milligrams per deciliter (mg/dL).

Day-5 Outcome Sampling

A peripheral venous sample of 1-2 mL was obtained on day 5 of life, timed close to 120 hours, with an allowable window of ±6 hours. TSB was measured and reported in milligrams per deciliter (mg/dL). If phototherapy was initiated before day 5, the highest recorded bilirubin value prior to treatment initiation was documented, and the neonate was classified as having developed significant hyperbilirubinemia within the study period.

Outcome definition

The primary analytic outcome was significant hyperbilirubinemia on day 5, defined as venous TSB ≥ 15 mg/dL measured at approximately 120 hours (allowable window ±6 hours). The day-5 threshold of TSB ≥ 15 mg/dL was selected a priori as a fixed analytic endpoint to evaluate predictive performance during the expected peak bilirubin window and was not intended to represent the AAP phototherapy threshold. Phototherapy, if initiated at any time during the first 5 days, was based on AAP 2022 [13] hour-specific thresholds for infants ≥ 35 weeks (gestational age and neurotoxicity risk-factor strata). Phototherapy initiation was documented as a clinical management outcome and did not redefine the study cut point.

Data management and statistical analysis

Data were entered into Microsoft Excel (Microsoft Corp., Redmond, WA, USA) and analyzed using IBM SPSS Statistics for Windows, Version 29.0 (Released 2022; IBM Corp., Armonk, NY, USA). Continuous variables were summarized as mean ± standard deviation or median with interquartile range, depending on distribution. Categorical variables were summarized as counts and percentages. Associations between early bilirubin values and the day-5 outcome were assessed using chi-square tests after categorizing bilirubin levels using clinically relevant cut points. ROC curves were constructed for cord bilirubin and 24-hour bilirubin to assess discrimination and identify optimal predictive cutoffs. Optimal cutoffs were selected from ROC coordinates using the Youden index (J = sensitivity + specificity − 1). AUC values are reported with 95% CI. Sensitivity, specificity, positive predictive value, and negative predictive value were calculated for selected thresholds.

Ethical considerations

Ethical approval was obtained from the institutional ethics committee prior to study initiation (IEC No.: ESICMC/SNR/IEC-S0245/04-2023, Dated: May 10, 2023). Written informed consent was obtained from mothers before enrolment.

## Results

A total of 200 term neonates were enrolled and followed through day 5 of life. Significant hyperbilirubinemia, defined as day-5 TSB ≥ 15 mg/dL, was observed in 77/200 neonates (38.5%), while 123/200 (61.5%) had day-5 TSB < 15 mg/dL. No neonate received phototherapy before the scheduled day-5 sampling. Baseline maternal and neonatal characteristics are summarized. Most mothers were younger than 30 years (69.5%). Gestational age was predominantly 38-39 weeks (89.5%). Birth weight most commonly ranged from 3.1 to 3.5 kg (55.0%). Normal vaginal delivery accounted for 64.5% of births, and O-positive was the most frequent neonatal blood group (53.5%) (Table 1).

**Table 1:** Baseline maternal and neonatal characteristics (N = 200) Categorical variables are presented as n (%). LSCS: lower segment cesarean section.

Variable	Category	n (%)
Maternal age (years)	<30	139 (69.5)
	≥30	61 (30.5)
Gestational age (weeks)	38	83 (41.5)
	39	96 (48.0)
	≥40	21 (10.5)
Neonatal sex	Male	96 (48.0)
	Female	104 (52.0)
Birth weight (kg)	2.5-3.0	76 (38.0)
	3.1-3.5	110 (55.0)
	>3.5	14 (7.0)
Parity	Para 1	123 (61.5)
	Para 2	53 (26.5)
	Para 3	24 (12.0)
Mode of delivery	Normal vaginal delivery	129 (64.5)
	LSCS	71 (35.5)
Neonatal blood group	O+	107 (53.5)
	B+	53 (26.5)
	A+	29 (14.5)
	AB+	11 (5.5)

Oxytocin exposure occurred in 86/200 deliveries (43.0%). The first feed was initiated within three hours in 123/200 neonates (61.5%), while 77/200 (38.5%) experienced a delay beyond three hours (Table 2).

**Table 2:** Intrapartum exposure and early feeding variables (N = 200) Categorical variables are presented as n (%).

Variable	Category	n (%)
Oxytocin exposure	Yes	86 (43.0)
	No	114 (57.0)
First feed delay	>3 hours	77 (38.5)
	≤3 hours	123 (61.5)

Cord bilirubin and day-5 TSB distributions are summarized. Cord bilirubin demonstrated moderate variability, whereas day-5 TSB showed a wider range, extending into clinically significant values (Table 3).

**Table 3:** Summary of bilirubin measurements (N = 200) Variables are presented as mean ± standard deviation with range. TSB: total serum bilirubin.

Measure	Mean ± SD	Minimum	Maximum
Cord bilirubin (mg/dL)	1.874 ± 0.55	0.8	3.5
Day-5 TSB (mg/dL)	13.57 ± 2.64	6.3	18.7

Most cord bilirubin values were in the 1.0-1.9 mg/dL category (64.5%), while 27/200 neonates (13.5%) had cord bilirubin ≥ 3.0 mg/dL. The distribution of the primary outcome is presented in Table 4.

**Table 4:** Distribution of bilirubin categories (N = 200) Variables are presented as n (%). TSB: total serum bilirubin.

Measure	Category	n (%)
Cord bilirubin (mg/dL)	<1.0	12 (6.0)
	1.0-1.9	129 (64.5)
	2.0-2.9	32 (16.0)
	≥3.0	27 (13.5)
Day-5 TSB (mg/dL)	<15	123 (61.5)
	≥15	77 (38.5)

The day-5 outcome distribution did not differ meaningfully by sex. The proportions were similar across both groups, and the association test was not significant (Table 5).

**Table 5:** Neonatal sex and day-5 outcomes (N = 200) Values are presented as n (%). Percentages are row percentages. Pearson chi-square test was applied. TSB: total serum bilirubin.

Sex	Day-5 TSB < 15 mg/dL, n (%)	Day-5 TSB ≥ 15 mg/dL, n (%)	Total (n)	p-value
Male	61 (63.5)	35 (36.5)	96	0.57
Female	62 (59.6)	42 (40.4)	104	

A strong gradient was observed across weight bands. The lowest weight band carried the largest event proportion. The association was statistically significant (Table 6).

**Table 6:** Birth weight and day-5 outcomes (N = 200) Values are presented as n (%). Percentages are row percentages. Pearson chi-square test was applied. TSB: total serum bilirubin.

Birth weight (kg)	Day-5 TSB < 15 mg/dL, n (%)	Day-5 TSB ≥ 15 mg/dL, n (%)	Total (n)	p-value
2.5-3.0	18 (23.7)	58 (76.3)	76	<0.001
3.1-3.5	98 (89.1)	12 (10.9)	110	
>3.5	7 (50.0)	7 (50.0)	14	

Cord bilirubin showed a clear stepwise increase in the proportion of neonates with day-5 TSB ≥ 15 mg/dL across increasing bilirubin categories. The highest cord bilirubin category (≥3.0 mg/dL) had the largest proportion of outcome events, with 18/27 neonates (66.7%) meeting the day-5 threshold. The association was statistically significant (Table 7).

**Table 7:** Cord bilirubin categories and day-5 outcomes (N = 200) Values are presented as n (%). Percentages are row percentages. Pearson chi-square test was applied. TSB: total serum bilirubin.

Cord bilirubin (mg/dL)	Day-5 TSB < 15 mg/dL, n (%)	Day-5 TSB ≥ 15 mg/dL, n (%)	Total (n)	p-value
<1.0	10 (83.3)	2 (16.7)	12	<0.001
1.0-1.9	91 (70.5)	38 (29.5)	129	
2.0-2.9	13 (40.6)	19 (59.4)	32	
≥3.0	9 (33.3)	18 (66.7)	27	

Cord bilirubin demonstrated strong discrimination for predicting day-5 significant hyperbilirubinemia. The suggested cutoff provided high sensitivity with moderate specificity. The ROC test result was statistically significant (Table 8).

**Table 8:** Receiver operating characteristic (ROC) summary for cord bilirubin predicting day-5 significant hyperbilirubinemia (TSB ≥ 15 mg/dL)

ROC parameter	Value
AUC	0.893
Standard error	0.024
95% CI for AUC	0.846-0.940
p-value	<0.001
Suggested cutoff (mg/dL)	1.75
Sensitivity (%)	89.7
Specificity (%)	69.9

A 24-hour bilirubin demonstrated good discrimination for predicting day-5 significant hyperbilirubinemia (AUC 0.88, 95% CI 0.83-0.93, p < 0.001). A cutoff of 5.80 mg/dL provided 88.0% sensitivity and 80.5% specificity. The negative predictive value was 96.0%, indicating that values below the cutoff were rarely followed by day-5 TSB ≥ 15 mg/dL, while the positive predictive value was 54.0%, supporting its role as an early risk stratification tool rather than a standalone treatment trigger (Table 9).

**Table 9:** Receiver operating characteristic (ROC) summary for 24-hour bilirubin predicting day-5 significant hyperbilirubinemia (TSB ≥ 15 mg/dL) PPV: positive predictive value, NPV: negative predictive value.

ROC parameter	Value
AUC	0.880
Standard error	0.026
95% CI for AUC	0.83-0.93
p-value	<0.001
Suggested cutoff (mg/dL)	5.80
Sensitivity (%)	88.0
Specificity (%)	80.5
PPV (%)	54.0
NPV (%)	96.0

## Discussion

This study addressed a clinically relevant question: whether cord blood bilirubin and 24-hour serum bilirubin can predict significant hyperbilirubinemia by day 5 among healthy term neonates. Both measures demonstrated strong discrimination and clinically useful operating characteristics, supporting their role in early risk stratification during the post-discharge peak window.

In this cohort, 38.5% of neonates met the prespecified analytic endpoint of day-5 TSB ≥ 15 mg/dL under universal prospective biochemical follow-up. This proportion should not be interpreted as the background population incidence of phototherapy-requiring jaundice. Cord bilirubin showed a clear stepwise association with the outcome, with increasing event proportions across rising cord bilirubin categories. This relationship was supported by ROC analysis, which demonstrated strong discrimination (AUC 0.893). A cutoff of 1.75 mg/dL yielded high sensitivity (89.7%) with moderate specificity (69.9%), a performance profile consistent with a screening threshold intended to minimize missed high-risk infants. The 24-hour bilirubin performed comparably (AUC 0.880), and a cutoff of 5.80 mg/dL provided 88.0% sensitivity and 80.5% specificity, with a high negative predictive value of 96.0%. This indicates that values below the threshold were rarely followed by day-5 TSB ≥ 15 mg/dL and supports the utility of the 24-hour measurement as an early rule-out marker. In addition, birth weight was significantly associated with the outcome, with higher risk concentrated in the 2.5-3.0 kg band, whereas sex was not associated with day-5 hyperbilirubinemia.

Agreement with the literature

The cord bilirubin findings are consistent with prior work reporting similar discrimination and comparable thresholds. Castillo et al. reported an AUC of approximately 0.89 for cord bilirubin in predicting clinically relevant hyperbilirubinemia outcomes, with a cutoff near 1.85 mg/dL [8]. The cutoff of 1.75 mg/dL identified in the present study lies within the range reported in other cohorts. Aktas et al. and Mittana and Arimilli reported cord bilirubin cutoffs between 1.67 and 1.95 mg/dL, with useful screening performance and high negative predictive value [15,16]. The stepwise gradient across cord bilirubin strata observed in this cohort is also aligned with stratified patterns described by Sun et al., where higher cord bilirubin categories were associated with higher rates of subsequent clinically significant jaundice [7].

The 24-hour bilirubin findings also align with established evidence supporting first-day bilirubin as an early screening tool. Alpay et al. and Spoorthi et al. reported that day-1 bilirubin thresholds close to 6 mg/dL provide high sensitivity and strong negative predictive value for later significant hyperbilirubinemia [10,12]. The cutoff of 5.80 mg/dL in the present study is concordant with that range. Carbonell Estrany et al. similarly highlighted the predictive value of early bilirubin measures in term neonates and emphasized their utility for early risk identification [11]. The association between lower birth weight and increased risk is consistent with the concept that smaller infants, even within term gestation, may have reduced physiological reserve and more vulnerable feeding transition, as also reported by Singla et al. [17].

Areas of divergence and possible explanations

Two findings warrant consideration. First, the event rate of 38.5% exceeds that reported in several term cohorts, where rates commonly range from approximately 10% to 20%. For example, Spoorthi et al. reported 10.3%, and Mittana and Arimilli reported 16.5% [12,16]. This difference may reflect complete follow-up through day 5, the use of a fixed threshold of 15 mg/dL as the endpoint, local feeding transition patterns, or surveillance intensity within the study setting. Each of these factors can influence observed incidence.

Second, meta-analytic evidence has suggested that higher cord bilirubin thresholds, often around 2.5-3.0 mg/dL, may improve specificity for predicting phototherapy need [9]. In contrast, the present study identified a lower threshold of 1.75 mg/dL to prioritize sensitivity. This trade-off is clinically defensible in contexts where follow-up is inconsistent, and the primary aim is to reduce missed high-risk infants. Importantly, unlike cohorts enriched for hemolysis, this study excluded ABO and Rh incompatibility, suggesting that prediction in this cohort reflects physiological jaundice dynamics rather than immune hemolysis. This may partly explain differences in specificity when compared with hemolysis-enriched samples, including studies focused on ABO incompatibility [6,18].

Study contribution

This study contributes to the existing literature by evaluating early bilirubin predictors in a defined cohort of healthy term appropriate-for-gestational-age (AGA) neonates rather than in higher-risk jaundice populations. First, the analysis used a prespecified day-5 outcome window centered around 120 hours, which corresponds to the usual bilirubin peak period in term neonates. Second, both cord bilirubin and 24-hour bilirubin were assessed within the same cohort, allowing direct comparison of their predictive performance. Third, the observed category-wise gradient in risk and the association with lower birth weight may assist follow-up planning within similar routine term populations. However, these findings are population-specific and should not be generalized to neonates excluded from the present study, including preterm infants, hemolytic jaundice, and other clinically high-risk groups.

Strengths

The prospective design supports temporal validity, and follow-up through day 5 reduces missed late peaks. Eligibility criteria were clearly defined, with exclusion of major hemolysis-related confounders. Laboratory methods were uniform across time points. ROC analysis was applied with clear reporting of discrimination and cutoff performance. The evaluated predictors are also feasible in routine care because they rely on widely available bilirubin measurements without specialized equipment or composite indices.

Limitations

The study was conducted at a single center, and external validation is required before adopting specific cutoffs across settings. The endpoint used a fixed TSB threshold of 15 mg/dL rather than hour-specific percentile-based nomograms, which may limit direct comparability with Bhutani-style risk zoning. Feeding adequacy was assessed clinically but not quantified in detail, limiting evaluation of effect modification by feeding practices. Finally, the relatively high event rate may reflect local care pathways or surveillance intensity, and generalizability to settings with structured post-discharge follow-up requires caution.

## Conclusions

In this prospective cohort of healthy term neonates, a clinically meaningful proportion developed day-5 significant hyperbilirubinemia. Both cord bilirubin at birth and the 24-hour bilirubin demonstrated strong discriminatory performance for the day-5 endpoint, with AUC values close to 0.9, indicating good overall classification ability. Clinically, the 24-hour measurement showed a very high negative predictive value, around the mid-90 percent range, supporting its use as an early rule-out marker to identify infants who are unlikely to develop significant hyperbilirubinemia and who may be suitable for routine follow-up. At the same time, higher early bilirubin values should be interpreted as risk flags that justify reinforced feeding support and earlier reassessment rather than automatic phototherapy, because treatment decisions remain hour-specific and guideline-based. Although two early measurements are pragmatic for discharge counselling and surveillance planning, these results do not establish that a combined dual time-point model improves prediction beyond either test alone. Limitations include the single-center design, exclusion of hemolysis-related jaundice, use of a prespecified day-5 threshold rather than an hour-specific treatment threshold as the analytic outcome, and limited measurement of feeding-related modifiers. External validation in independent cohorts is required before adopting these markers as standardized decision rules across diverse settings.
